# NONO Maintains SREBP‐Regulated Cholesterol Biosynthesis via RNA Binding in Neuroblastoma

**DOI:** 10.1096/fj.202403267RR

**Published:** 2025-09-16

**Authors:** Song Zhang, Hayley Ingram, Jack Cooper, Alina Naveed, Stefan G. Kathman, Garrett L. Lindsey, Tao Liu, Charles S. Bond, Jamie I. Fletcher, Benjamin F. Cravatt, Archa H. Fox

**Affiliations:** ^1^ School of Human Sciences The University of Western Australia Crawley Western Australia Australia; ^2^ Department of Chemistry The Scripps Research Institute La Jolla California USA; ^3^ Children's Cancer Institute Australia Randwick New South Wales Australia; ^4^ Centre for Childhood Cancer Research UNSW Sydney Kensington New South Wales Australia; ^5^ School of Molecular Sciences The University of Western Australia Crawley Western Australia Australia; ^6^ Children's Cancer Institute, Lowy Cancer Research Centre, School of Clinical Medicine, UNSW Medicine & Health UNSW Sydney Kensington New South Wales Australia; ^7^ Harry Perkins Institute of Medical Research, QEII Medical Centre Nedlands Western Australia Australia

**Keywords:** cholesterol, DBHS, expression, neuroblastoma, RNA binding

## Abstract

High‐risk neuroblastoma is associated with upregulation of cholesterol biosynthesis through increased expression of sterol regulatory element—binding protein (SREBP). NONO, a multifunctional nuclear RNA binding protein, is an established oncogene in neuroblastoma and can stabilize SREBP in breast cancer. Hence, here we addressed the unexplored question of NONO regulation of SREBP in neuroblastoma. We show NONO knockdown reduces cholesterol in neuroblastoma patient‐derived tumor cell lines and high‐risk neuroblastoma KELLY cells. NONO knockdown also reduces mRNA and protein expression of SREBP family members in KELLY cells. RNA‐seq of NONO knockdown confirmed cholesterol synthesis pathway genes are downregulated. Further, only overexpression of NONO wild‐type, rather than NONO mutant lacking the RNA recognition motif 1, could elevate SREBP levels after endogenous NONO knockdown, revealing the importance of NONO RNA binding activity. Finally, (R)‐SKBG‐1, a small molecule that modulates the RNA binding activity of NONO, hence altering its subnuclear distribution, significantly decreased cholesterol levels and SREBP target gene expression in KELLY cells. These results lend weight to manipulating NONO RNA binding as a potential therapeutic avenue for treating aggressive neuroblastoma.

## Graphical abstract

We identified that NONO plays a role in maintaining sterol regulatory element—binding protein (SREBF)‐regulated cholesterol biosynthesis in high‐risk neuroblastoma. A small molecule ligand (R)‐SKBG‐1 that modulates the RNA binding activity of NONO significantly decreased the SREBF‐cholesterol pathway, highlighting the importance of manipulating RNA binding as a potential therapeutic avenue for treating the disease.

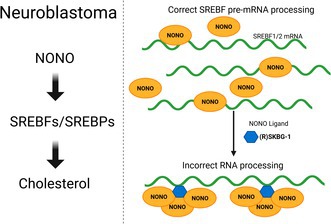

## 1 Introduction

Neuroblastoma arises within the first 5 years of life, and accounts for roughly 10% of all pediatric cancers [1]. Neuroblastoma derives from primordial neural crest cells that are defective in migration and maturation/differentiation [1]. With high clinical and biological heterogeneity, neuroblastoma is challenging to treat [1, 2]. Increased cholesterol and/or lipids provide dividing cells with essential components for rapid plasma membrane synthesis, as well as acting as alternative energy sources, allowing for cancer cells to regulate their biophysical properties that enhance their proliferative and migratory capabilities [3, 4]. A large transcription factor and epigenetic regulatory network is associated with neuroblastoma, and transcriptional upregulation of cholesterol and lipid metabolic pathways has been explored and attributed to high‐risk neuroblastoma states [5]. A more recent study proposed total cholesterol levels in serum as a prognostic marker of neuroblastoma, where patients who had relapsed and/or succumbed to the disease displayed a significant increase in total cholesterol serum levels [6].

Drivers of lipid and cholesterol synthesis are being intensively investigated within the context of cancer, with the maturing appreciation of proliferation dependency on these factors. A key transcription factor family controlling lipid and cholesterol synthesis is Sterol Regulatory Element Binding Protein (SREBP), which exhibits elevated expression in thyroid cancer, negatively impacting the efficacy of cancer treatments, and is associated with poor prognosis in breast cancer [7, 8]. SREBPs exist in three isoforms within mammalian cells. SREBP1‐a and ‐c collectively regulate both lipid and cholesterol synthesis, while SREBP2 mainly mediates cholesterol synthesis [9]. SREBPs regulate lipid and cholesterol synthesis by binding to sterol‐regulated elements (SREs) within target gene promoters [9]. SREBP expression, subcellular localization, and stability are controlled by interactions with other proteins [10]. For example, in breast cancer, SREBP protein expression levels are positively correlated with the expression of the RNA‐binding protein Non‐POU Domain Containing Octamer Binding (NONO) and NONO stabilizes SREBP1 to help drive breast cancer cell proliferation [11].

NONO is a member of the Drosophila Behavior Human Splicing (DBHS) protein family, which is collectively involved in gene regulation at transcriptional, post‐transcriptional, and translational levels [12, 13, 14, 15]. Distinct regions of NONO have differing roles in interactions with RNA, DNA, and protein. For example, the NOPS (NonA/paraspeckle) domain is implicated in protein–protein interaction and dimerization, while the canonical RNA recognition motif (RRM1) is essential for RNA binding. High expression of NONO is associated with poor patient outcomes in neuroblastoma, suggesting an oncogenic role [16, 17]. Our previous RNA‐seq experiment after NONO knockdown (KD) in neuroblastoma revealed many differentially expressed genes, but one major significant pathway for downregulated genes was cholesterol synthesis and metabolism [16]. In addition, we previously showed that the RNA binding activity of NONO plays a critical role in the regulation of pre‐mRNA processing and gene expression in neuroblastoma, but did not specifically examine factors involved in cholesterol biosynthesis [16].

Exploration of the mechanisms regulating lipid and cholesterol synthesis specific to neuroblastoma is limited. Moreover, modulation of RNA binding with small molecules is an emerging therapeutic modality. Therefore, in this study, we aimed to investigate the role and mechanism of NONO in the cholesterol synthesis pathway in high‐risk neuroblastoma and hypothesized that RNA binding capacity is critical for NONO's effects in this pathway. In endogenous NONO knockdown, total cholesterol and SREBP1/2 expression were investigated in KELLY and patient‐derived high‐risk neuroblastoma cell lines, alongside overexpression experiments using NONO wild‐type and NONO mutants lacking RNA binding capability (ΔRRM1). Additionally, total cholesterol and SREBP target gene expression were compared between cells incubated with a small molecule ligand that specifically targeted the RNA binding site of NONO and inactive molecule controls. Collectively, our data demonstrate a role for NONO in maintaining SREBP‐dependent cholesterol levels in neuroblastoma, via its ability to bind RNA.

## 2 Materials and Methods

### 2.1 Cell Culture

Human neuroblastoma KELLY cells were grown in Gibco RPMI 1640 (Thermo Fisher Scientific, 11835055) supplemented with 10% Foetal Bovine Serum (FBS) and 100 U/mL Penicillin–Streptomycin (Pen/Strep). Four high‐risk neuroblastoma patient‐derived early passage cell lines (COG‐N‐496, COG‐N‐415, COG‐N‐440 and COG‐N‐519 cell lines) were grown in Iscove's Modified Dulbecco's Medium (Thermo Fisher Scientific, 12440061) supplemented with 20% FBS, 4 mM L‐Glutamine (Thermo Fisher Scientific, 25030081) and 1 × Insulin‐Transferrin‐Selenium (Thermo Fisher Scientific, 41400045). These patient‐derived cell lines were obtained from the Childhood Cancer Repository, Texas Tech University Health Sciences Center in Lubbock, TX, USA, and a complete list of mutated genes from the matched patient‐derived xenografts can be found via the Pediatric Preclinical Testing Consortium (https://pedcbioportal.kidsfirstdrc.org/study/summary?id=pptc). For cholesterol assays, FBS was maintained at 1% in FBS‐reduced growth medium. Cells were trypsinized for routine passaging with Gibco Tryple Express (ThermoFisher, 12604021) and cultured at 37°C in an incubator supplied with 5% CO_2_.

### 2.2 Transfection

The siRNAs used in the study were Silencer Select Negative Control No. 1 siRNA (Thermofisher 4390844), Silencer Select siRNA NONO s9612 (Thermofisher 4392422) and Silencer Select siRNA NONO s9613 (Thermofisher, 4392420). Plasmids including EYFP control, EYFP‐NONO_WT, and EYFP‐NONO_∆RRM1 (aa67‐141 deletion) were previously made [16] and contained the siRNA resistance site corresponding to siRNA NONO s9612. Lipofectamine RNAiMAX (ThermoFisher, 13778150) and Lipofectamine 3000 (ThermoFisher, L3000015) were the transfection reagents used in this study. All transfection mixtures were made up in serum‐reduced Gibco Optimem (ThermoFisher, 11058021), as per the manufacturer's instructions. For siRNA‐only transfections, 25 nM of siRNAs and Lipofectamine RNAiMAX were used. Sequential transfections for overexpression experiments were performed using 10 nM of siRNAs and Lipofectamine RNAiMAX to reduce the endogenous NONO, followed by 1.25 μg of plasmid DNA and Lipofectamine 3000 on the next day. The cells were collected 2 days after the final transfection.

### 2.3 Drug Treatment

Small molecules (S)‐SKBG‐1 and (R)‐SKBG‐1 were dissolved in DMSO at 20 mM and further diluted to a final concentration of 10 μM. Cells were incubated with DMSO, (S)‐SKBG‐1 or (R)‐SKBG‐1 for 48 h before imaging or harvesting.

### 2.4 Cholesterol Assay

KELLY cells and patient‐derived cell lines were seeded at 5 × 10^5^ in 6‐well plates and grown in FBS‐reduced growth media for at least 3 days before harvesting. Cells were scraped and washed in PBS before resuspension in PBS, with an aliquot for total protein measurement via Bradford assays. To the remaining cell suspension, 200 μL of Chloroform:Isopropanol:NP‐40 (7:11:0.1) was added, and samples were sonicated at room temperature for 5–10 min. After centrifuging extracts at 20 000× *g* for 10 min at room temperature, the liquid organic phase was transferred and air dried at 50°C to remove organic solvent. Total cholesterol content was determined by Cholesterol Assay Kit (Biovision, K603‐100) according to the manufacturer's instructions. Dried lipids were dissolved in 150 μL of Cholesterol Assay Buffer by sonicating for 5 min. 30 μL of each standard and sample in duplicate was added to a 96‐well plate. A reaction mix was further added in each well, containing Cholesterol Assay Buffer, Cholesterol Probe, Cholesterol Enzyme, and Cholesterol Esterase. After mixing for 2 min, the plate was incubated for 60 min at 37°C protected from light, then fluorescence was measured in a microplate reader (CLARIOstar Plus, BMG Labtech).

### 2.5 Protein Extraction and Western Blot

Cells were lysed with RIPA buffer (150 mM NaCl, 25 mM Tris pH 7.5, 1% sodium deoxycholate, 0.1% SDS, 1% IPEGAL CA‐630, 1 × Protease Inhibitor). In a Bradford assay, 10 μL of each sample and BSA standard in duplicate (Promega, R3961) were added to a 96‐well plate. 200 μL of diluted Protein Assay Dye Reagent (Bio‐Rad, 500‐0006) was then added to each sample/standard, and absorbance was measured in a microplate reader (CLARIOstar Plus, BMG Labtech). Protein samples were mixed with SDS gel‐loading buffer and heated at 70°C for 10 min. Samples and Precision Plus Protein All Blue Prestained Protein Standards (Bio‐Rad, 1610373) were loaded onto Mini‐PROTEAN TGX Pre‐Cast gels (Bio‐Rad, 4561086). Gels were run in Tris/Glycine buffer (Bio‐Rad, 1610771) at 200V, and membrane transferred using Trans‐Blot Turbo Mini Nitrocellulose Transfer packs (Bio‐Rad, 1704158). Primary antibodies including SREBP1 (rabbit polyclonal, Abcam, ab28481) and SREBP2 (rabbit polyclonal, Abcam, ab30682) were diluted 1:500 in 5% milk PBST. The secondary horseradish‐peroxidase conjugated antibody goat anti‐rabbit IgG H&L HRP (Abcam, ab97051) was diluted 1:5000. Luminata Crescedo Western HRP substrate (Merck, WBLUR0100) was added, and blot images were acquired by Bio‐Rad Chemidoc. Bio‐Rad Imagelab Version 5.2 was used to quantify total protein levels and the intensity of the protein chemiluminescent bands. The relative intensity of chemiluminescent bands was normalized to the amount of total protein in each sample lane, and the sizes of chemiluminescent bands determined in relation to the standards ladder bands.

### 2.6 RNA Isolation and Real Time RT‐qPCR

Cells were lysed with Nucleozol (Macherey‐Nagel, 740404). To each sample, molecular‐grade water was added, and tubes were vortexed for 15 s. All samples were incubated at room temperature for 5 min and then heated to 55°C and shaken at 1000 rpm for 10 min. After centrifuging, supernatant was transferred, and an equal volume of isopropanol was added with 1 μL of GlycoBlue co‐precipitant (ThermoFisher, AM9516) to aid pellet visualization. Samples were incubated at −20°C for 1 h and centrifuged at 14 000× *g* for 10 min at 4°C. The RNA pellets were washed with 70% ethanol and resuspended in molecular‐grade water. RNA concentration was determined with a ThermoFisher Nanodrop 2000. The QuantiTect Reverse Transcription (RT) kit (Qiagen, 205314) was used for reverse transcribing the isolated RNA samples. For each RNA sample, 2 μL of gDNA Wipeout was added and incubated for 2 min at 42°C. A mastermix was made on ice at a dilution of 4:1:1 with 5 × Quantiscript RT buffer: RT Primer mix: Quantiscript Reverse Transcriptase. The RT mastermix was added to each sample. Samples were incubated for 30 min at 42°C, then 3 min at 95°C. Real‐time qPCRs were performed in the Rotor‐Gene Q real‐time PCR cycler (Qiagen). A PCR reaction consisted of SensiMix SYBR No‐ROX (Bioline, QT650‐20), 250 nM of forward and reverse primers (Integrated DNA Technologies, Table 1), molecular‐grade water, and cDNA. U6 spliceosomal RNA (U6) was used as a reference gene, and relative mRNA expression level was presented using the 2^−ΔΔCt^ method.

**TABLE 1:** A list of primers.

Primer	Direction	5′—3′
U6	Forward	CTCGCTTCGGCAGCACA
	Reverse	AACGCTTCACGAATTTGCGT
NONO	Forward	AGGAAGGATTCAAGGGAACC
	Reverse	GCATGGCACCTCTGTTGTT
SREBF1	Forward	ACTCGCTGCTTCTGACAGCC
	Reverse	TCAGTGCCCACCACCAGATCC
SREBF2	Forward	CCTCAACCTCAAACTCAGCTGC
	Reverse	CTGCTGCTGAATGGTGACCG
ACAT2	Forward	CAGAAAGCTGGCCATTTTGA
	Reverse	CATGGCGAGGAAACTCATCT
CYP51A1	Forward	TGCAGATTTGGATGGAGGTTTCAG
	Reverse	TGATTTCCCGATGAGCTCTGTCC
DHCR7	Forward	GAGCTCCACAGCCATGTGA
	Reverse	GGCAGATGTCAATGGTCTTCA
DHCR24	Forward	TCTGCACTGCTTACGAGCTG
	Reverse	AGGACCAGGGTACGGCATAGAAC
FDFT1	Forward	AGGAAGAGAGTTCTGGCCTCAAG
	Reverse	CAAGTCAATATTCTCCGGCTTAGC
HMGCR	Forward	ATCCGTTTCCAGTCCAGGTC
	Reverse	GCTAGAATCTGCATTTCAGGG
HSD17B7	Forward	TGCTGGGATCATGCCTAATCCAC
	Reverse	GCCTTCAGCTGTGGAGAACATATG
NSDHL	Forward	CCAATGAGAGACCAAGTCGCAC
	Reverse	CACCGATCACTGTGCATCTCTTG

### 2.7 Immunofluorescence and Image Analysis

Cells grown on glass coverslips (Schott, G405‐15) were fixed using 4% paraformaldehyde in PBS for 10 min at room temperature. Coverslips were permeabilized in freshly made 1% Triton X‐100 for 15 min at room temperature. For sequential double immunofluorescence, epitope detection was firstly conducted with a primary monoclonal mouse antibody against NONO at a 1:500 dilution in PBST for 1 h at room temperature. After three PBST washes, coverslips were incubated with an anti‐mouse FITC conjugated secondary antibody (Jackson Laboratories, 115‐095‐072) at a 1:250 dilution in PBST for 1 h at room temperature. After extensive PBST washes, cells were then incubated with a monoclonal mouse antibody against SC35 (Sigma‐Aldrich, anti‐splicing factor S4045) at a 1:100 dilution in PBST overnight at 4°C, followed by the incubation with an anti‐mouse CY5 conjugated secondary antibody (Jackson Laboratories, 115‐175‐166) at a 1:250 dilution for 1 h at room temperature. Coverslips were counterstained with DAPI for 5 min at room temperature. For dual immunofluorescence and RNA Fluorescence In Situ Hybridization (FISH), after immunofluorescence with NONO, coverslips were then hybridized overnight with FISH probes against human NEAT1 middle segment with Quasar 670 Dye (NEAT1_2, Stellaris, VSMF‐2251‐5), a well‐defined structural scaffold for paraspeckles, at 37°C according to the manufacturer's instructions.

Coverslips were mounted on microscope slides using VECTASHIELD Mounting Medium (Abacus, H‐1000) and clear nail varnish. All images were acquired on a Nikon C2+ confocal microscope and NIS‐Elements Advanced (4.0) software (Nikon) using a 60× objective for NONO and NEAT1 or a 100× objective for NONO and SC35. Acquisition parameters were kept consistent, and intensity thresholds were set the same for samples within each experiment.

## 3 Data Analysis

All data has been presented as mean ± SD. The number of biological replicates is shown in figure legends. Homogeneity of variance was tested using Levene's test. One‐way analysis of variance (ANOVA) was used to compare group means when variances were equal, followed by Tukey's Honestly Significant Difference (HSD) test for post hoc comparisons. Welch's ANOVA was used for unequal variances, with the Games‐Howell test applied for post hoc comparisons when significant. Data presented was interpreted as *p < 0.05; **p < 0.01 and ***p < 0.001. All graphs were generated in R.

## 4 Results

**FIGURE 1:**
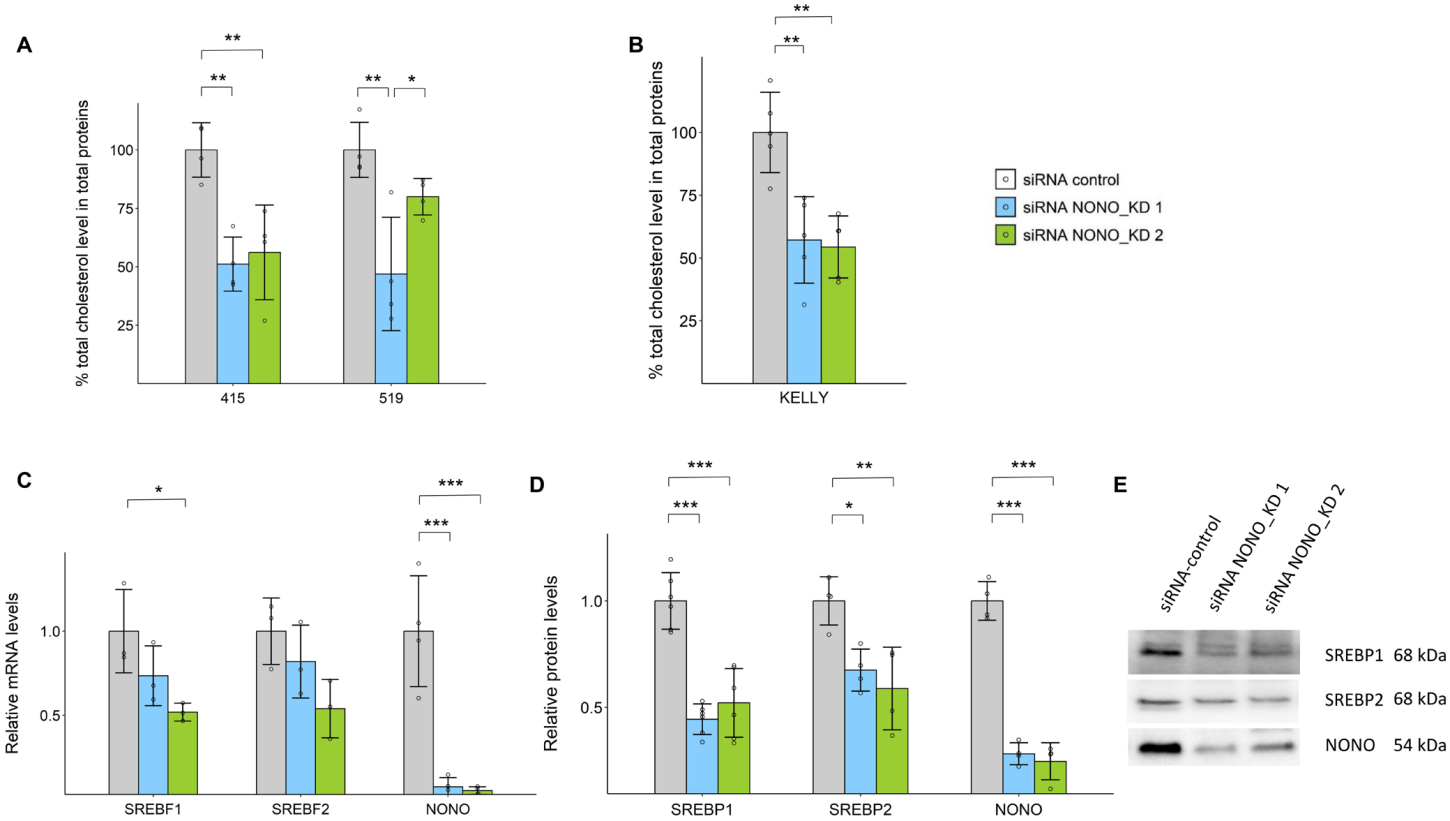
NONO Knockdown results in reduced cholesterol, SREBF1 mRNA and SREBP protein levels. (A) Total cholesterol levels between control and NONO KD siRNAs in 415 and 519 high‐risk neuroblastoma patient‐derived cell lines (*n* = 4). (B) Total cholesterol levels between control and NONO KD siRNAs in KELLY cells (*n* = 5). (C) Relative SREBF1 (*n* = 3), SREBF2 (*n* = 3) and NONO (*n* = 4) mRNA levels via RT‐qPCR between control and NONO KD siRNAs in KELLY cells. (D) Western blot quantitation analysis of SREBP1 (*n* = 6), SREBP2 (*n* = 4) and NONO (*n* = 4) protein levels between control and NONO KD siRNAs in KELLY cells. (E) Representative western blot images for SREBP1, SREBP2 and NONO proteins in (D). Data are expressed as mean ± SD. *p < 0.05; **p < 0.01 and ***p < 0.001.

To investigate the role of NONO in cholesterol synthesis in aggressive neuroblastoma, we measured total cholesterol levels after NONO KD using two different siRNAs in a variety of neuroblastoma cell lines (Figures 1 and S1). We observed NONO KD led to a significant reduction in total cholesterol levels in several recent patient‐derived high‐risk neuroblastoma cell lines (lines 415 and 519, Figure 1A), and the well‐established and well‐studied high‐risk neuroblastoma KELLY cell line (Figure 1B). In line with the known heterogeneity of neuroblastoma, this NONO‐cholesterol dependency was not universal to all recent patient‐derived neuroblastoma lines (Figure S1). For all subsequent experiments, KELLY cells were used.

**FIGURE 2:**
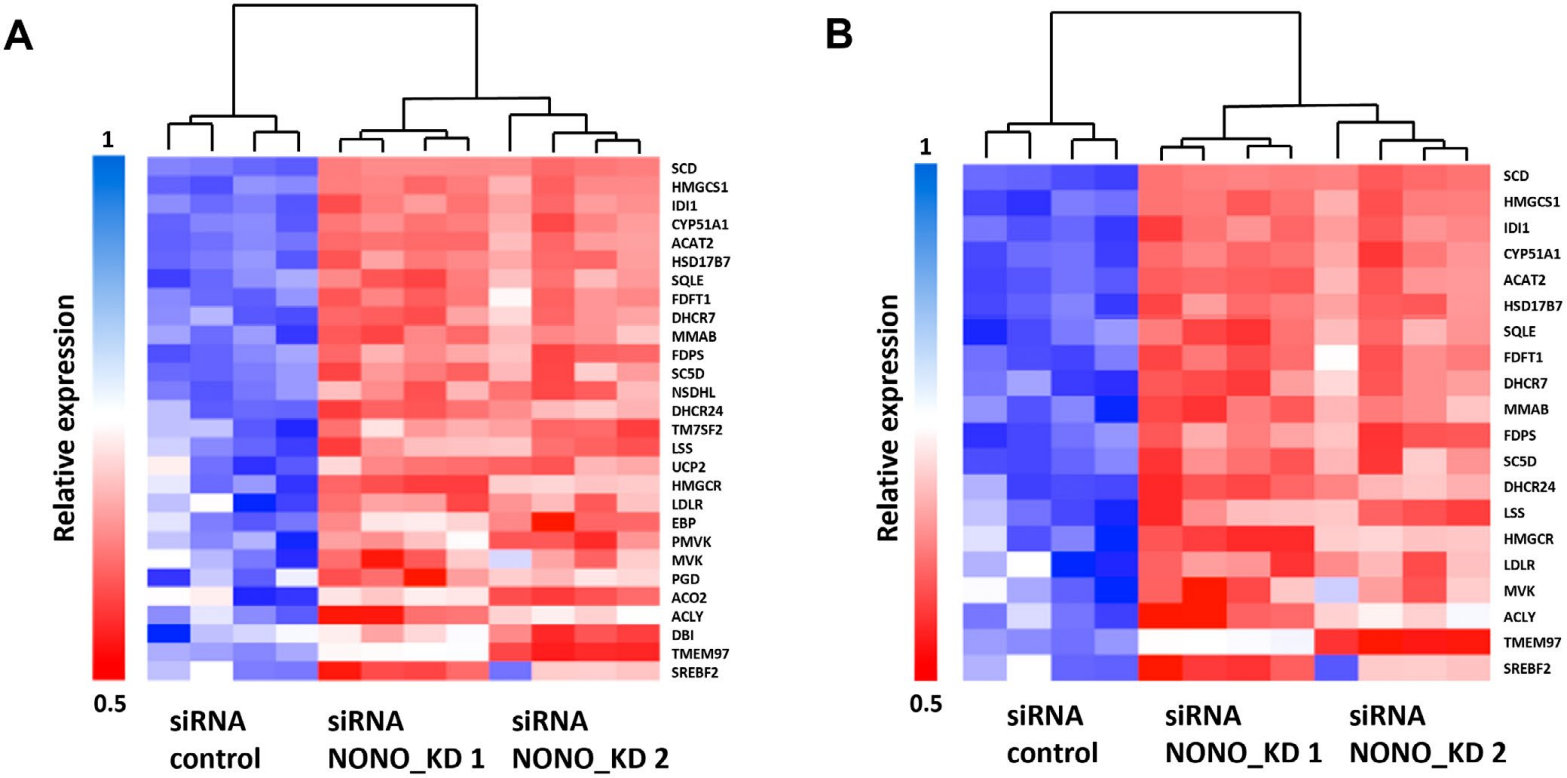
NONO KD results in decreased expression of the target genes of both SREBP1 (A) and SREBP2 (B) based on RNA‐seq data in KELLY cells (*n* = 4). Heatmaps depicting gene expression levels between control and NONO KD siRNAs.

The ablation of NONO significantly reduced NONO mRNA and protein levels (Figure 1C–E). SREBP1 and SREBP2, encoded by the SREBF1 and SREBF2 genes, are master transcription factors that mediate the expression of a host of genes required for cholesterol and lipid synthesis [7, 8]. We next examined the effect of NONO on the expression of both key transcription factors. NONO knockdown reduced SREBF1 and SREBF2 mRNA levels (Figure 1C) as well as SREBP1 and SREBP2 protein levels (Figure 1D,E). We next re‐examined our prior published RNA‐seq data from NONO KD in KELLY cells that had revealed broad downregulation of genes in the cholesterol biosynthesis pathway. Here, we specifically examined the differential gene expression of the gene sets that are downstream targets of either SREBP1 (Figure 2A) or SREBP2 (Figure 2B) and observed in both cases a significant reduction in the expression of these SREBP1/2 target genes following NONO KD.

**FIGURE 3:**
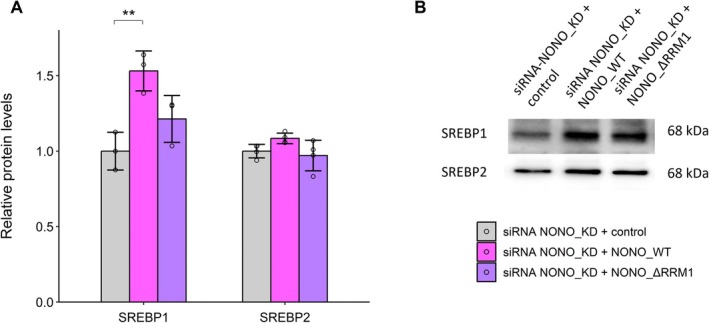
Overexpression of NONO_WT, but not NONO_∆RRM1, increases SREBP1 protein levels following KD of endogenous NONO. (A) Quantitation of western blotting data showing relative SREBP1 (*n* = 3) and SREBP2 (*n* = 4) protein levels in KELLY cells transfected sequentially with NONO KD siRNA, and then plasmid expressing siRNA‐resistant control protein (YFP only), plasmid expressing YFP fused NONO_WT protein, or plasmid expressing YFP fused NONO_∆RRM1 protein. (B) Representative western blot images for SREBP1 and SREBP2 proteins in (A). Data are expressed as mean ± SD. **p < 0.01.

We also previously carried out and reported transcriptome‐wide Cross linking‐Immunoprecipitation (CLIP) experiments of NONO in KELLY cells that showed extensive NONO binding to the 5′ ends of oncogenic pre‐mRNA [16]. Re‐examining this data for SREBF1 and 2, a similar pattern was also detected for NONO binding to the SREBF1 and SREBF2 pre‐mRNA (Figure S2), suggesting NONO exerts its effects on SREBP1/2 via binding to their pre‐mRNA, SREBF1/2. To explore this further, we turned to a previously characterized NONO RNA binding mutant, lacking the first RNA binding domain (NONO_ΔRRM1) [16]. To minimize the effects of endogenous NONO, we first knocked down endogenous NONO using siRNA and then overexpressed siRNA‐resistant plasmids expressing either wild‐type NONO (YFP‐NONO_WT) or NONO lacking RRM1 (YFP‐NONO_∆RRM1). With western blotting, we observed over‐expression of NONO_WT, but not NONO_∆RRM1, could elevate SREBP1 protein levels (Figure 3A,B), suggesting that NONO maintenance of SREBP levels is at least partly dependent on its RNA binding activity. Of note, when over‐expressed in KELLY cells, the RNA binding mutant localizes to a small number of spherical foci, unlike NONO wildtype, that localizes to many smaller, finer, broadly distributed nuclear foci [16].

**FIGURE 4:**
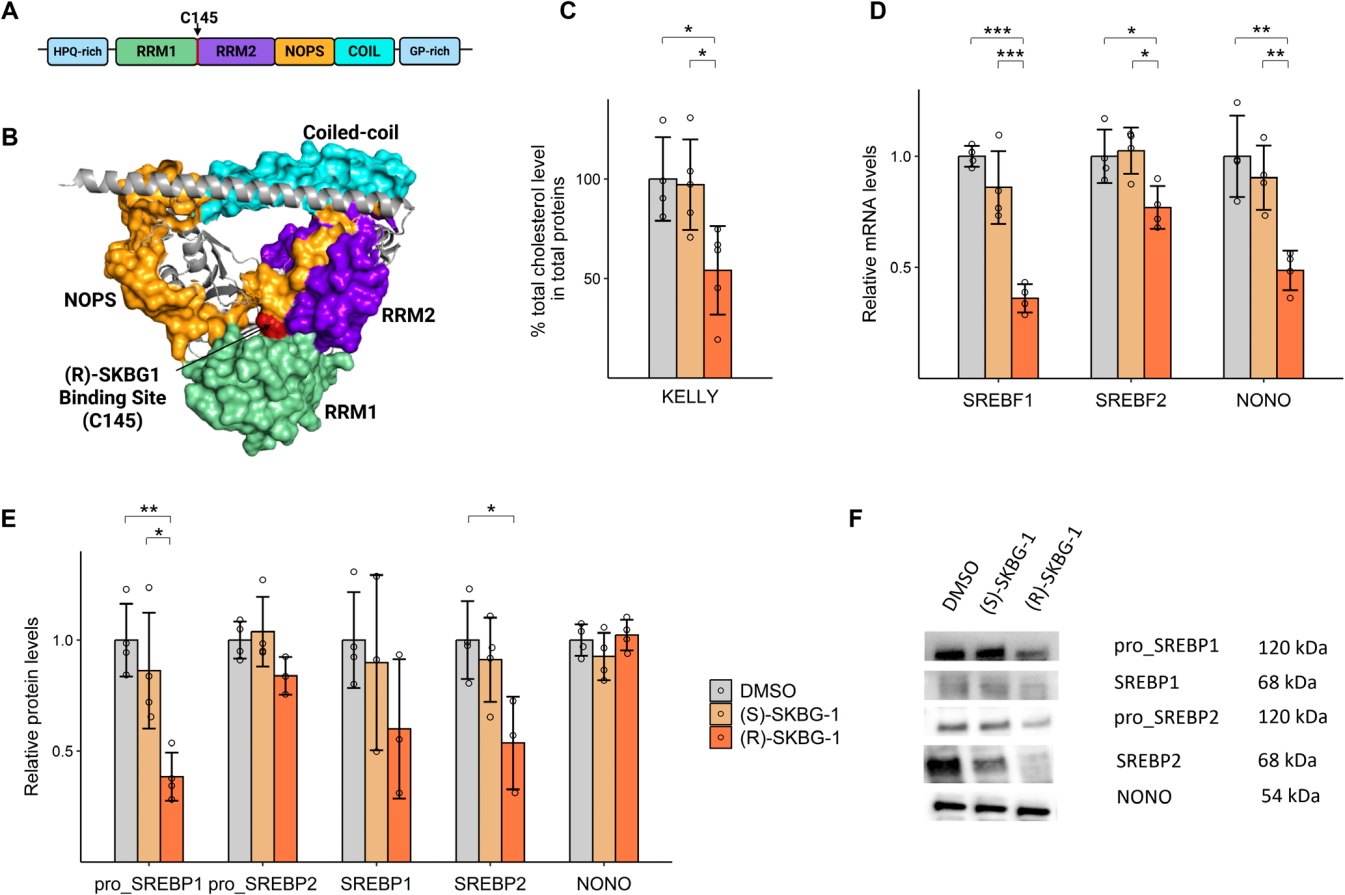
(R)‐SKBG‐1 reduces cholesterol levels, mRNA levels of SREBF1, SREBF2 and NONO as well as protein abundance of pro_SREBP1 and SREBP2. (A) Domain structure of NONO with 5′ and 3′ low complexity regions (light blue), RNA recognition motifs RRM1 (green) and RRM2 (purple), (R)‐SKBG‐1 compound binding site at position C145 (red), NonA/paraspeckle (NOPS) (orange) and coiled‐coil (cyan). (B) Heterodimer crystallography structure of PSPC1 (gray) and NONO with domains consistent with colors as above (excludes low complexity regions). Model created with PyMol using crystal structure from the Protein Data Bank (ID: 3SDE). (C) Total cholesterol levels between KELLY cells treated with DMSO, (S)‐SKBG‐1 or (R)‐SKBG‐1 (*n* = 5). (D) Relative SREBF1, SREBF2 and NONO mRNA levels via RT‐qPCR between DMSO, (S)‐SKBG‐1 and (R)‐SKBG‐1 in KELLY cells (*n* = 4). (E) Western blot quantitation analysis of pro‐SREBP1 (precursor form), SREBP1 (mature), pro‐SREBP2, SREBP2 and NONO protein levels between DMSO, (S)‐SKBG‐1 and (R)‐SKBG‐1 in KELLY cells (*n* = 4). (F) Representative western blot images for pro‐SREBP1, SREBP1, pro‐SREBP2, SREBP2 and NONO proteins in (E). Data are expressed as mean ± SD. *p < 0.05; **p < 0.01 and ***p < 0.001.

**FIGURE 5:**
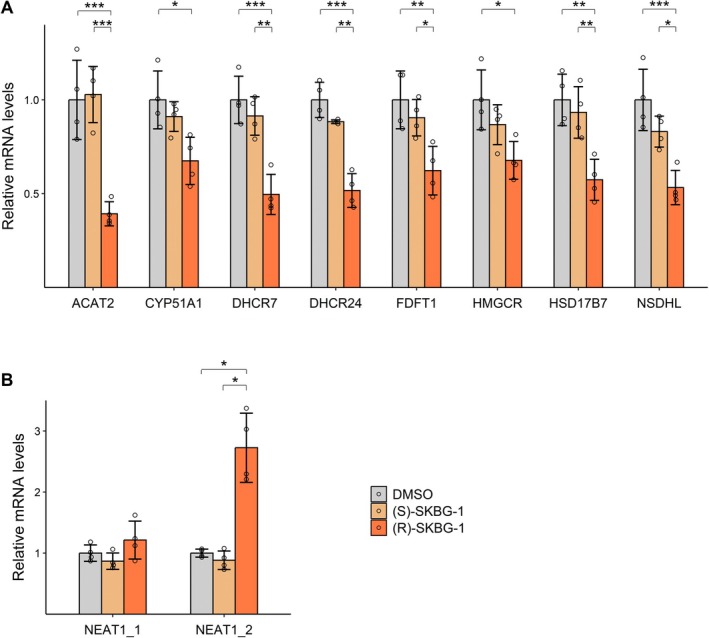
(R)‐SKBG‐1 reduces mRNA levels of SREBP target genes but increases NEAT1_2 RNA expression. (A) (R)‐SKBG‐1 results in decreased mRNA levels of SREBP1 and SREBP2 target genes including ACAT2, CYP51A1, DHCR7, DHCR24, FDFT1, HMGCR, HSD17B7 and NSDHL in KELLY cells, as measured with RT‐qPCR (*n* = 4). (B) (R)‐SKBG‐1 has no effect on NEAT1_1, but increases NEAT1_2 expression (*n* = 4). Data are expressed as mean ± SD. *p < 0.05; **p < 0.01 and ***p < 0.001.

To further delineate the importance of the RNA binding function of NONO on cholesterol synthesis, we turned to a small molecule that was recently shown to bind NONO and alter the dynamics of its RNA binding [18]. We incubated cells with the small molecule ligand (R)‐SKBG‐1, which specifically binds a cysteine proximal to RRM1 to form a covalent bond with NONO (Figure 4A,B). Although (R)‐SKBG‐1 binds adjacent to RRM1, prior data indicate it does not inhibit RNA binding, but rather modulates the dynamics of RNA binding, increasing the association of NONO with its RNA substrates in a ‘RNA trapping’ mechanism [18]. Conveniently, (S)‐SKBG‐1, an inactive enantiomer of (R)‐SKBG‐1, cannot bind to NONO, and therefore acts as an additional negative control alongside DMSO, the chemical used to dissolve both small molecules. Applying these chemicals to KELLY cells, we observed (R)‐SKBG‐1, but not the controls, led to a decrease in total cholesterol levels (Figure 4C). We used RT‐qPCR to show that (R)‐SKBG‐1 reduced mRNA levels of SREBF1, SREBF2, and NONO (Figure 4D). Interestingly, western blots showed (R)‐SKBG‐1 treatment did not alter NONO protein levels, although there was decreased abundance of SREBP1 precursor (pro_SREBP1) and mature SREBP2 (Figure 4E,F). Further, the mRNA expression of several SREBP1 and SREBP2 target genes, as measured by RT‐qPCR, was decreased after incubation with (R)‐SKBG‐1, including ACAT2, CYP51A1, DHCR7, DHCR24, FDFT1, HMGCR, HSD17B7, and NSDHL (Figure 5A).

One of the top RNA bound by NONO in KELLY cells is the long noncoding RNA (lncRNA), NEAT1_2, that is required, with NONO, to form subnuclear paraspeckle foci [16]. NONO coats NEAT1_2 and stabilizes it to form paraspeckles. This NEAT1_2 binding activity of NONO is in contrast to its role in binding oncogenic pre‐mRNA, where processing to facilitate efficient mRNA production from pre‐mRNA is the key activity. We previously showed that increasing NEAT1_2/paraspeckles in KELLY cells could sequester NONO away from its oncogenic pre‐mRNA targets, thereby inhibiting NONO's oncogenic role in pre‐mRNA processing in neuroblastoma [19]. Hence, we carried out RT‐qPCR analysis that showed (R)‐SKBG‐1 treatment increased NEAT1_2 RNA expression without altering a control paraspeckle‐independent NEAT1_1 isoform transcribed from the same locus (Figure 5B). Thus, overall, a small molecule that alters NONO RNA binding dynamics reduced SREBF mRNA, SREBP protein, and their target gene mRNA expression but increased NEAT1_2 expression, suggesting tweaking NONO RNA binding dynamics gives different outcomes for different RNA substrates.

**FIGURE 6:**
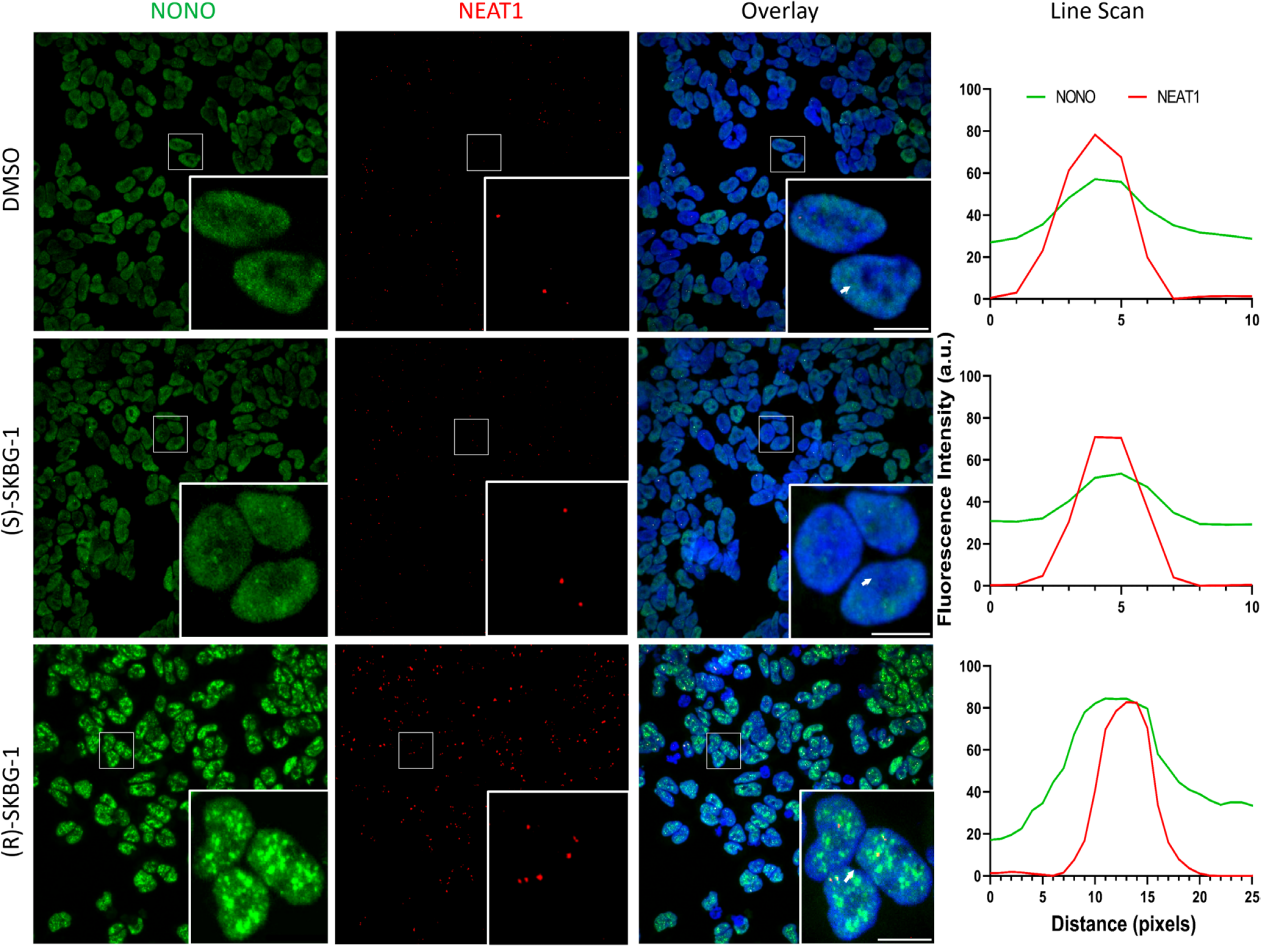
(R)‐SKBG‐1 treatment results in increased NONO foci and increased paraspeckles in KELLY cells. Fluorescence micrograph images of representative cells subject to NONO immunofluorescence (green), NEAT1_2 RNA FISH (red), and cell nuclei via DAPI (blue). KELLY cells were treated with either DMSO (top), (S)‐SKBG‐1 (middle) or (R)‐SKBG‐1 (bottom) prior to staining. Line scans correspond to the positions and directions of the arrows. Scale bar: 10 μm.

**FIGURE 7:**
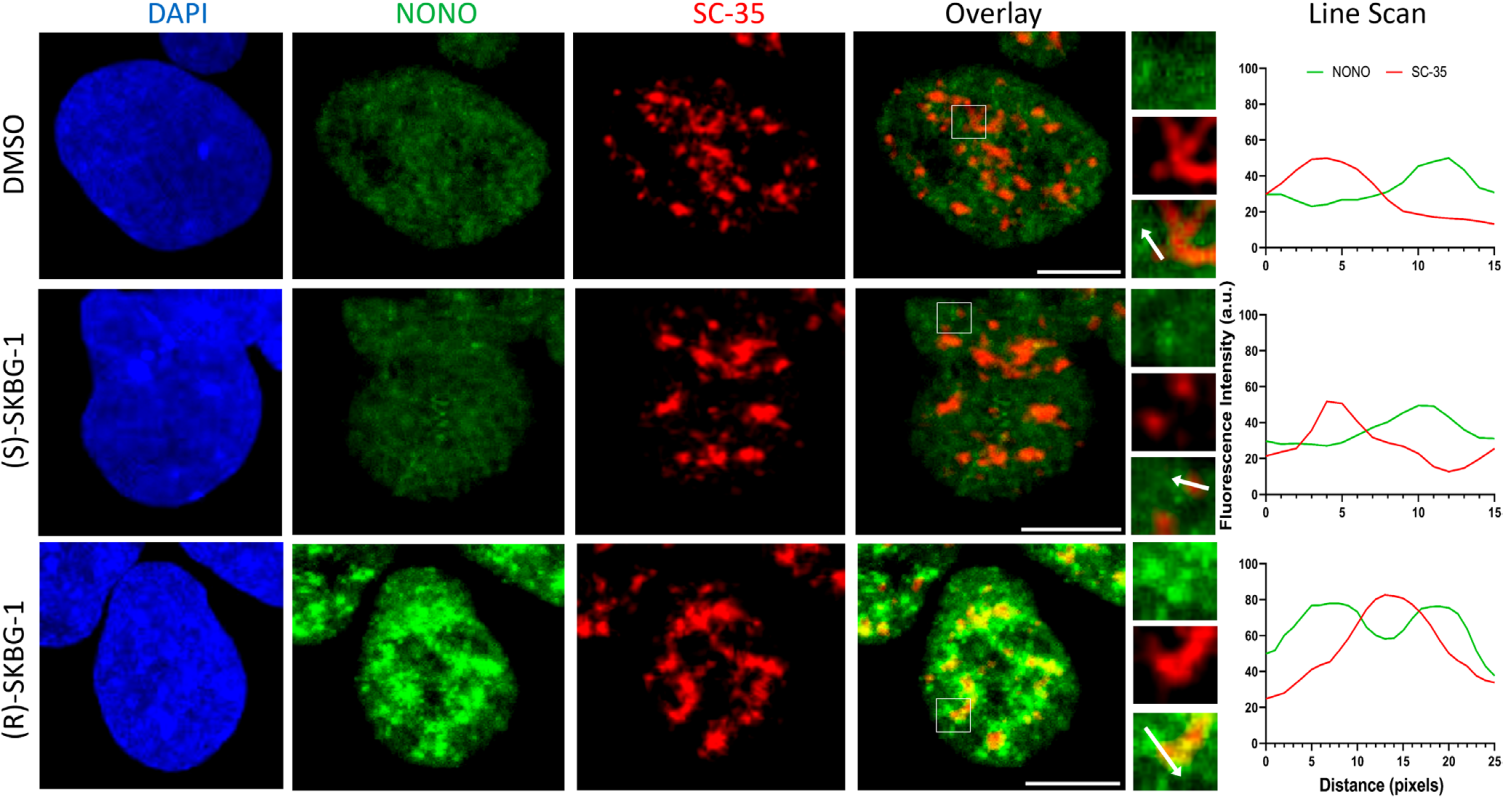
(R)‐SKBG‐1 treatment results in increased NONO foci that surround nuclear speckles. Fluorescence micrograph images of representative cells subject to NONO immunofluorescence (green), SC35 immunofluorescence (red) and cell nuclei via DAPI (blue). KELLY cells were treated with either DMSO (top), (S)‐SKBG‐1 (middle) or (R)‐SKBG‐1 (bottom) prior to staining. Line scans correspond to the positions and directions of the arrows. Scale bar: 5 μm.

Next, we examined the sub‐nuclear distribution of endogenous NONO following (R)‐SKBG‐1 treatment, as distinct NONO sub‐nuclear distribution in aggressive‐subtype neuroblastoma cells is linked to its RNA binding and regulatory activity [16]. Specifically, unlike many other cell types, NONO displays much smaller and more numerous fine nuclear puncta in neuroblastoma cells that are associated with its pre‐mRNA binding. Using immunofluorescence against endogenous NONO and DAPI nuclear co‐staining, after treatment with (R)‐SKBG‐1, (S)‐SKBG‐1 and DMSO, we observed much larger and more distinct NONO nuclear puncta in (R)‐SKBG‐1 treatment compared to the controls (Figure 6, lower panel). We have previously demonstrated that paraspeckles are co‐localized with a subset of NONO immunofluorescence signals [19, 20]. The incubation with (R)‐SKBG‐1 significantly increased paraspeckles, consistent with the increased NEAT1_2 RNA levels, which retain and increase co‐localization with accumulated NONO foci (Figure 6). Finally, we examined the relationship of NONO foci after (R)‐SKBG‐1 treatment with nuclear speckles. Recently, it was demonstrated that highly transcribed genes with higher splicing efficiency and RNA processing requirements are found in close proximity to nuclear speckles [21]. Strikingly, the aggregated NONO foci observed after (R)‐SKBG‐1 treatment localize in a distinct cloud surrounding SC35‐labeled nuclear speckles (Figure 7). Taken together, these data suggest modulation of NONO RNA binding by (R)‐SKBG‐1 leads to altered sub‐nuclear distribution and lowered SREBP levels, leading to cholesterol reduction.

## 5 Discussion

In this study we identified that the RNA binding protein NONO plays a role in maintaining SREBP‐regulated cholesterol biosynthesis in high‐risk neuroblastoma. In the absence of NONO, SREBP mRNA and protein levels significantly decreased, as did expression of downstream target genes of both SREBP1 and SREBP2. Further, SREBP protein levels could not be elevated by the addition of NONO mutants lacking RNA binding capability (ΔRRM1) compared to the wild‐type. Additionally, total cholesterol and SREBP target gene expression were decreased in cells incubated with a small molecule ligand (R)‐SKBG‐1 that modulated NONO's RNA binding capacity and sub‐nuclear distribution, but did not lower total NONO protein levels. This highlights that NONO is important for maintaining SREBP expression in the cholesterol synthesis pathway, which we speculate is likely through its RNA binding function.

### 5.1 RNA Binding Capacity Is Essential for NONO in Maintaining Cholesterol Synthesis in Neuroblastoma

Here we show that the ablation of NONO significantly reduced SREBP‐regulated total cholesterol levels in patient‐derived clinical cell lines and high‐risk neuroblastoma KELLY cells. In high‐risk neuroblastoma patients, high expression of NONO is associated with poor patient outcomes and prognosis [16, 17]. High levels of cholesterol‐associated proteins and/or transcripts are also linked to worse clinical outcomes in neuroblastoma and other cancers such as carcinomas and ovarian cancers [5, 6, 22, 23]. Thus, our findings propose a potential link between high expression of NONO and cholesterol biosynthesis in neuroblastoma.

NONO is a multifunctional protein that interacts with RNA, DNA, and protein to regulate gene expression at the transcriptional, post‐transcriptional, and translational levels. In NONO KD experiments, the broad inhibition of NONO expression may contribute to the reduced expression of SREBF mRNAs, SREBP proteins, and their target genes via many different mechanisms such as protein–protein, protein–RNA, or DNA binding. However, in the current study, we focused on the impact of NONO RNA binding ability on the regulation of SREBF–cholesterol biosynthesis. The overexpression of NONO_WT but not NONO_ΔRRM1 enhanced SREBP1 protein levels, suggesting a role of RNA binding in regulating SREBP levels, despite that equivalent expression levels of NONO_WT and ΔRRM1 are not guaranteed. The incubation with a small molecule specifically modulating RNA binding of NONO decreased the SREBF‐cholesterol pathway without altering NONO protein abundance, providing strong evidence that RNA‐binding capacity is critical for NONO to regulate SREBPs and their target genes to maintain cholesterol synthesis in neuroblastoma. This was supported by prior CLIP data showing NONO binds to SREBF pre‐mRNAs extensively within introns, especially at the first intron.

In contrast with our findings, a previous study focusing on breast cancer showed NONO protein interacted with SREBP‐1a protein via a conserved Y267 residue in the NONO NOPS domain [11]. The authors also used embryonic kidney (HEK293) cells to show NONO knockdown increased ubiquitination of SREBP‐1a, suggesting the role of NONO was to prevent ubiquitination of SREBP. Further, these authors reported NONO KD, or overexpression, did not change SREBF1 mRNA levels [11]. In contrast, our findings show in several patient‐derived and KELLY neuroblastoma cell lines, NONO KD significantly reduced both mRNA and protein levels of SREBP1 and 2. Whilst the exact mechanism of how NONO RNA binding activity affects SREBF mRNA expression is unclear, it is possible that NONO nuclear condensates, which regulate mRNA processing, splicing, and gene regulation at the transcriptional and post‐transcriptional levels, play a role [12, 13, 14, 15, 16]. Thus, there may exist several different mechanisms, either via protein–protein or protein‐RNA interactions, explaining how NONO regulates SREBP. Future experiments to tease out if these mechanisms are exclusive or additive in different cancer types would be beneficial.

### 5.2 Targeting Cholesterol Pathways as a Potential Therapeutic Route

Abnormal lipid phenotypes have been attributed to mesenchymal transition that promotes metastatic spread often seen in highly aggressive cancers [24]. Additionally, cholesterol homeostasis has been suggested to mediate tumor progression by dampening immune cell responses [25]. Establishing the relationship between lipid/cholesterol synthesis and cancer has been explored in many subtypes including breast, prostate, colorectal, and endometrial cancers, where the latter established a link between cholesterol homeostasis and the tumor microenvironment [24, 26]. As cholesterol metabolism plays a role in cancer cell proliferation and migratory capabilities, it is clinically relevant to investigate these pathways further for potential treatment options in neuroblastoma and other cancers.

Poor patient outcomes have been linked with elevated levels of cholesterol and cholesterol‐associated genes in a variety of tumor cell types, including neuroblastoma [6, 22, 23]. Other cancers such as acute myeloid leukemia have seen promising results in combination therapy that targets the cholesterol pathway alongside standard chemotherapy [27]. Targeting cholesterol synthesis pathways helps to overcome radio‐ and chemo‐sensitivity, as well as manipulating the tumor microenvironment to reduce metastases in some cancers [28, 29, 30, 31]. These treatments often rely on statin drugs that can have varied benefits among cancer subtypes depending on their cells individual reliance on components within the lipid and cholesterol pathways, pointing to the need for more targeted approaches [32]. However, precise targeting of the cholesterol synthesis pathway is challenging due to the broad list of components regulating the pathway, lack of knowledge of molecular mechanisms, and desire to prevent toxicity in non‐cancer cells. Nevertheless, for neuroblastoma, the need to find better treatments is especially evident [33].

Our results using (R)‐SKBG‐1, a small molecule that modulates NONO‐RNA interaction, represent a useful starting point for the development of NONO‐RNA targeted anti‐cholesterol neuroblastoma treatments [18]. The dysregulation of NONO RNA binding ability induced by (R)‐SKBG‐1 may lead to decreased SREBF‐cholesterol pathway by preventing NONO from dissociating from its RNA substrates. This ‘trapping’ of NONO on the RNA likely inhibits the correct processing of these pre‐mRNAs. Moreover, it is likely that NONO targeted pre‐mRNAs are highly transcribed and require enhanced splicing regulation. This is because, following (R)‐SKBG‐1 treatment, the NONO foci increased specifically in the region surrounding nuclear speckles. Nuclear speckles were recently demonstrated to be surrounded by highly transcribed and splicing regulated genes [21]. Interestingly, the enhanced co‐localization between NONO and paraspeckles in (R)‐SKBG‐1 treated cells indicates that NONO ligands may stabilize NONO binding to NEAT1_2, leading to the accumulation of NONO with upregulated paraspeckles. A previous study reported lower cell viability associated with increased NEAT1_2/paraspeckles in KELLY cells, proposing a possibility that increased sequestration of NONO by enlarged paraspeckles might be in part responsible for the reduction in cell viability [19].

(R)‐SKBG‐1 is useful due to NONO's substantial stereoselectivity for this active compound and not the inactive stereo‐isomer (S)‐SKBG‐1, which was the case in a very small subset out of over 5000 proteins previously investigated [18]. Additionally, the site specificity of (R)‐SKBG‐1 for C145 in NONO required for compound binding, and therefore the unusual trapping mechanism, is unique to this DBHS protein and is absent in paralogues SFPQ and PSPC1, preventing their usual compensatory actions in response to disruptions in NONO functionality [18]. These qualities provide vital controls for investigating the specific pharmacological effects of (R)‐SKBG‐1 on NONO, and more broadly, the SREBP‐mediated cholesterol pathway neuroblastoma cells are reliant upon. If (R)‐SKBG‐1 or its future derivatives were a viable option, it will be important to determine the toxicity that modulating NONO‐RNA binding has on healthy tissues. Thus, overall, our study opens the door to further investigations targeting NONO‐RNA binding activity as a cholesterol‐reducing agent in neuroblastoma.

## Author Contributions

S.Z. designed research, conducted experiments, performed data analyses, interpreted results, and wrote the manuscript; H.I. conducted experiments, performed data analyses, interpreted results, and wrote the manuscript; J.C. performed data analyses and interpreted results; A.N. conducted experiments and interpreted results; S.G.K., C.S.B., J.I.F., and B.F.C. contributed to study conception and results interpretation; A.H.F. was the senior author and contributed to study conception, design, results interpretation, and manuscript writing. All authors reviewed the results and approved the final manuscript.

## Conflicts of Interest

The authors declare no conflicts of interest.

## Supporting information


**Figure S1:** NONO KD has no effect on total cholesterol levels in two additional high‐risk neuroblastoma patient‐derived cell lines (496 and 440) (*n* = 2).
**Figure S2:** NONO binds predominantly to the first introns of SREBF1 and SREBF2 genes based on PAR‐CLIP data. Genome browser graph‐based tracks are configured to show the display height in pixels from 0 to 1000. Small boxes represent exons and UTRs, and lines for introns. Arrows on lines indicate the direction of transcription. Red circles highlight the first introns with relatively strong NONO binding compared with other regions in the same genes.

## Data Availability

The data that support the findings of this study are available upon request from the corresponding author.
