# Supplementary material for: NONO Maintains SREBP‐Regulated Cholesterol Biosynthesis via RNA Binding in Neuroblastoma
Source: FASEB J. 2025 Sep 16;39(18):e71051. doi: 10.1096/fj.202403267RR (PMC12439068; doi:10.1096/fj.202403267RR)
Supplement: Supplementary file 1 — Figure S1: NONO KD has no effect on total cholesterol levels in two additional high‐risk neuroblastoma patient‐derived cell lines (496 and 440) (n = 2). Figure S2: NONO binds predominantly to the first introns of SREBF1 and SREBF2 genes based on PAR‐CLIP data. Genome browser graph‐based tracks are configured to show the display height in pixels from 0 to 1000. Small boxes represent exons and UTRs, and lines for introns. Arrows on lines indicate the direction of transcription. Red circles highlight the first introns with relatively strong NONO binding compared with other regions in the same genes. [file FSB2-39-e71051-s001.pdf]

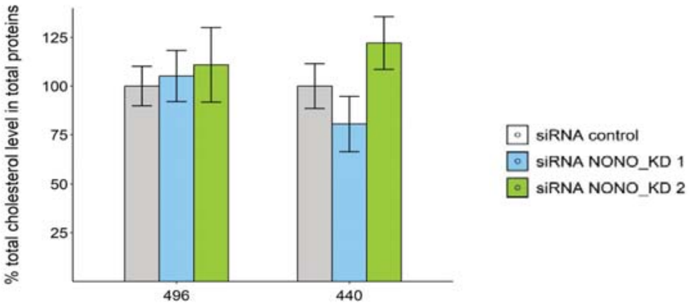

Supplementary Figure 1. NONO KD has no effect on total cholesterol levels in two additional high-risk neuroblastoma patient-derived cell lines (496 and 440) (n=2).

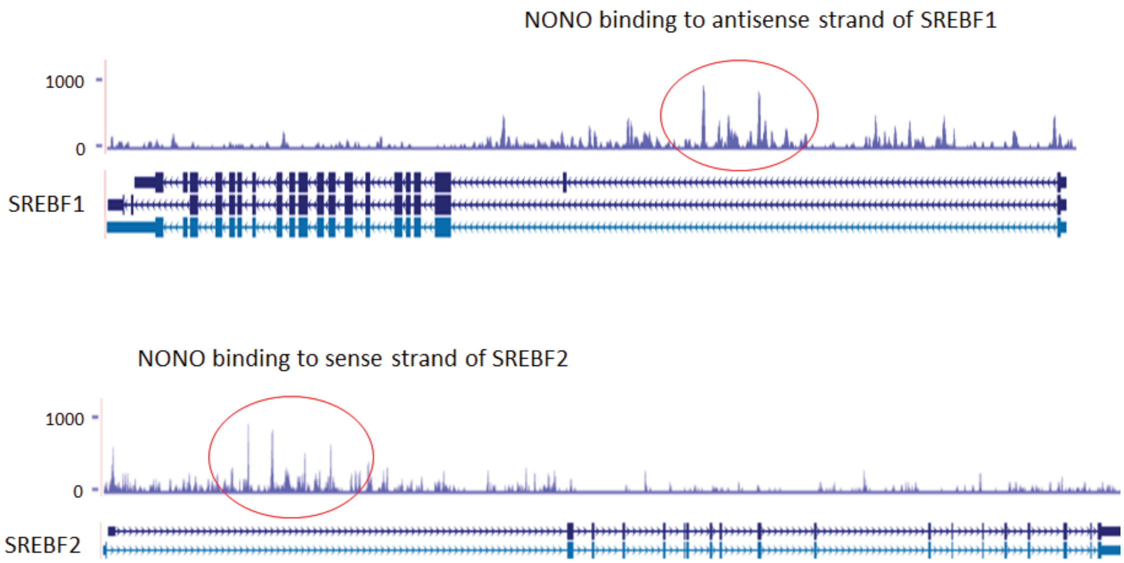

Supplementary Figure 2. NONO binds predominantly to the first introns of SREBF1 and SREBF2 genes based on PAR-CLIP data. Genome browser graph-based tracks are configured to show the display height in pixels from 0 to 1000. Small boxes represent exons and UTRs, and lines for introns. Arrows on lines indicate the direction of transcription. Red circles highlight the first introns with relatively strong NONO binding compared with other regions in the same genes.
